# Smoking among pregnant women in Cantabria (Spain): trend and determinants of smoking cessation

**DOI:** 10.1186/1471-2458-7-65

**Published:** 2007-04-27

**Authors:** Silvia Palma, Rocio Pérez-Iglesias, Rosa Pardo-Crespo, Javier Llorca, Marcial Mariscal, Miguel Delgado-Rodríguez

**Affiliations:** 1 Division of of Preventive Medicine and Public Health, University of Jaen, Jaen, Spain; 2 Division of Preventive Medicine and Public Health, University of Cantabria, Santander, Spain

## Abstract

**Background:**

Cantabria (Spain) has one of the highest prevalence of smoking among women of the European Union. The objectives are to assess the trend of smoking during pregnancy in a five-year period and the determinants of smoking cessation during pregnancy in Cantabria.

**Methods:**

A 1/6 random sample of all women delivering at the reference hospital of the region for the period 1998–2002 was drawn, 1559 women. Information was obtained from personal interview, clinical chart, and prenatal care records. In the analysis relative risks and 95% confidence intervals were estimated. Multivariable analysis was carried out using stepwise logistic regression.

**Results:**

Smoking prior to pregnancy decreased from 53.6% in 1998 to 39.4% in 2002. A decrease in smoking cessation among women smoking at the beginning of pregnancy was observed, from 37.3% in 1998 to 20.6% in 2002. The mean number of cigarettes/day (cig/d) before pregnancy remained constant, around 16 cig/d, whereas a slight trend to increase over time was seen, from 7.7 to 8.9 cig/d. In univariate analysis two variables favoured significantly smoking cessation, although they were not included in the stepwise logistic regression analysis, a higher education level and to be married. The logistic regression model included five significant predictors (also significant in univariate analysis): intensity of smoking, number of previous pregnancies, partner's smoking status, calendar year of study period (these four variables favoured smoking continuation), and adequate prenatal care (which increased smoking cessation).

**Conclusion:**

The frequency of smoking among pregnant women is very high in Cantabria. As smoking cessation rate has decreased over time, a change in prenatal care programme on smoking counseling is needed. Several determinants of smoking cessation, such as smoking before pregnancy and partner's smoking, should be also addressed by community programmes.

## Background

Cantabria, on the Cantabrian sea (northern Spain), has the highest frequency of smoking in Spanish women and one of the highest of the European Union, 35.6%, four points above the next area, the Basque region [1]. This can imply a high prevalence of smoking during pregnancy in our region, although no data are available. Smoking cessation during pregnancy is strongly recommended because of the adverse effects of tobacco on the newborn [2,3]. Reduction of smoking frequency among pregnant women has been reported in many countries [4-6], although the change during the last decade has been small in several areas [7]. Nevertheless, some women continue smoking during pregnancy, despite counseling on stop smoking in prenatal care. The assessment of the determinants of smoking cessation can contribute to improve the efficacy of prenatal care programmes. The main objectives of this report are to analyse the evolution of smoking among pregnant women in a five-year period and to assess the determinants of smoking cessation in a European community.

## Methods

The reference population was that of the region of Cantabria, northern Spain. The study period was from 1 April 1998 to 30 November 2002. Pregnant women were selected from those delivering at the University Hospital Marques de Valdecilla between 1 April 1998 and 30 November 2002 if they lived in Cantabria, the hospital's referral area. The hospital ethics committee authorised this observational study, and oral informed consent was sought from every eligible woman. During the study period, a random sample representing one-sixth of all women delivering at the hospital was drawn: all the women delivering on five days of each month, randomly selected in advance using the random number generator of a statistical program, were asked to participate. Twenty eight women declined to participate, yielding 1559 women in the study population.

The data were obtained from a personal interview, carried out within the three days after delivery, clinical charts and prenatal care records. We asked for tobacco consumption before and during pregnancy; all smokers before pregnancy continued smoking at the beginning of pregnancy (before they were aware of pregnancy). Therefore smoking prior to pregnancy and smoking at the beginning of pregnancy convey the same information. Information was obtained on the next variables: mother's vital data (age at pregnancy, race, education level, marital status, socioeconomic class, occupation), obstetric history (parity, abortions), previous adverse perinatal outcomes, conditions during pregnancy (infections, hypertension, diabetes mellitus, other obstetric conditions), prescribed and over-the-counter drugs, lifestyle (alcohol consumption, smoking), prenatal care (number of visits, date of first visit); and on the newborn (gestational age, anthropometric measures, Apgar score). Social class were coded in five main levels (ranging from I-highest- to V-lowest level-) according to the classification of the Spanish Society of Epidemiology [8], which is similar to the Black Report [9]. Prenatal care utilization was measured using the Kessner index [10]. This classification of prenatal care takes into account the month prenatal care began, the number of prenatal visits, and the duration of pregnancy; it differentiates three levels of care: adequate, intermediate, and inadequate.

In statistical analysis, the χ^2 ^test was used to assess changes in categorical variables during the study period; if a variable was continuous one-way analysis of variance was applied. The Mantel-Haenszel extension of χ^2 ^was used to ascertain the statistical significance of a trend for proportions. In bivariate analysis of smoking cessation, relative risks (RRs) and their 95% confidence intervals (CIs) were estimated; a RR > 1 indicates that a variable favours smoking cessation, whereas a RR < 1 denotes that a variable favours smoking continuation. To know the independent predictors smoking cessation, we developed a logistic regression model using a forward stepwise procedure. Variables with *p*-values lower than 15 percent were allowed in the final model. Analysis was carried out with the statistical package Stata 8/SE (College Station, Texas, USA).

## Results

The evolution of several characteristics of pregnant women throughout the study period is shown in table 1. Maternal age increased from 1998 to 2002 (*p *< 0.001). The proportion of primiparous women did not change. Although there was a significant difference in the frequency of an education level higher than high school, no clear trend was observed. Marital status, employment out home, and social class were roughly similar throughout the study period. Adequacy of prenatal care, measured by the Kessner index, clearly improved during the study period (*p *< 0.001). The frequency of smoking at the beginning of pregnancy steadily decreased from 1998 to 2002 (*p *< 0.001), whereas the average number of cigarettes/day in smokers did not change significantly during the study period. The frequency of smoking during the whole pregnancy during the study period remains stable, although an absolute decrease of 5.2% from 1999 to 2002 was appreciated.

**Table 1:** Evolution for the study period of several population variables.

	**Year**					
**Variable**	**1998** **(n = 315)**	**1999** **(n = 316)**	**2000** **(n = 302)**	**2001** **(n = 306)**	**2002** **(n = 320)**	***P*-value**

Age in years, mean ± SD	28.6 ± 5.0	29.6 ± 5.3	29.9 ± 4.9	29.9 ± 4.5	30.1 ± 4.9	< 0.001^a^
Primiparous, n (%)	135 (42.9)	137 (43.4)	140 (46.4)	130 (42.5)	141 (44.1)	0.603^b^
Education higher than secondary school, n (%)	181 (57.5)	155 (49.1)	121 (40.1)	121 (39.5)	180 (56.2)	< 0.001^b^
Married, n (%)	279 (88.6)	292 (92.4)	274 (90.7)	278 (90.8)	295 (92.2)	0.367^b^
Employment out home, n (%)	161 (51.1)	173 (54.7)	144 (47.7)	146 (47.7)	149 (46.6)	0.164^b^
Social class I-II, n (%)	41 (13.0)	37 (11.7)	49 (16.2)	37 (12.1)	36 (11.2)	0.227^b^
Kessner index: adequate prenatal care, n (%)	122 (38.7)	99 (31.3)	95 (31.5)	128 (41.8)	159 (49.7)	< 0.001^b^
Smokers prior to pregnancy, n (%)	169 (53.6)	163 (51.6)	132 (43.7)	135 (44.1)	126 (39.4)	< 0.001^b^
No. cig/d, mean ± SD	14.5 ± 7.6	14.9 ± 8.7	15.9 ± 8.6	17.1± 11.1	16.0 ± 8.9	0.207^a^
Smoking during the whole pregnancy, n (%)	106 (33.7)	115 (36.4)	108 (35.8)	104 (34.0)	100 (31.2)	0.367^b^

a One-way analysis of variance

b Mantel-Haenszel trend test

The history of smoking of pregnant women who were smokers at the beginning of pregnancy is detailed in table 2. The frequency of smoking cessation during pregnancy diminished from 37.3% in 1998 to 20.6% in 2002 (*p *< 0.001). The proportion of smokers that reduced tobacco use also increased, mainly from the year 1998 to 2000 (*p *< 0.001), whereas the proportion of those not changing their habit was similar along the study period. There were four women that increased smoking during pregnancy; they had received intermediate prenatal care. Pregnancy was unplanned in three of them and the four women rejected their pregnancy (they did not accept it).

**Table 2:** History of smoking during pregnancy in women smokers at the beginning of pregnancy.

**History of smoking**	**Year**					
	**1998** **(n = 169)**	**1999** **(n = 163)**	**2000** **(n = 132)**	**2001** **(n = 135)**	**2002** **(n = 126)**	***P*-value**

Cessation, n (%)	63 (37.3)	48 (29.4)	24 (18.2)	31 (23.0)	26 (20.6)	< 0.001^a^
Continuing smoking, n (%)	106 (62.7)	115 (70.6)	108 (81.8)	104 (77.0)	100 (79.4)	< 0.001^a^
^*c*^*Reduction in smoking, n (%)*	*92 (54.4)*	*101 (62.0)*	*100 (75.8)*	*97 (71.9)*	*88 (69.8)*	*< 0.001*^*a*^
^*c*^*No change or increase*^d^, *n (%)*	*14 (8.3)*	*14 (8.6)*	*8 (6.0)*	*7 (5.1)*	*12 (9.6)*	*0.563*^*a*^
No. cig/d, mean ± SD	7.7 ± 5.6	7.3 (4.4	6.9 (5.0	8.3 (5.0	8.9 (5.9	0.038b

a Mantel-Haenszel trend test

b One-way analysis of variance

c These are subgroups of women continuing smoking during pregnancy and the percentages are referred to the total number of women shown at the head of the column.

d Four women increased tobacco smoking

The association between several characteristics of women and smoking cessation is displayed in table 3. These analyses are based on smokers at the beginning of pregnancy (n = 725). Variables favouring significantly smoking cessation were a higher education level, to be married, and an adequate prenatal care (measured by the Kessner index). On the opposite side, the variables contributing significantly to keep smoking were to have had a previous pregnancy, the intensity of smoking before pregnancy, partner's smoking status, and the calendar year of the study. Variables unrelated to smoking cessation were maternal age, social class, change in alcohol drinking during pregnancy, and employment outside home. A stratified analysis by the calendar year of the determinants of smoking cessation was also carried out; no major differences during the study period were observed, apart from the lost of statistical significance due to the reduction of sample size; these results are not shown.

**Table 3:** Variables related to smoking cessation during pregnancy (based on 725 women smoking at the beginning of pregnancy).

Variables		Total	**Smoking cessation**	
		N	n (%)	RR (95% CI)

Age (years)	≥31	285	75 (26.3)	1.00 (0.72–1.38)
	26–30	281	75 (26.7)	1.01 (0.73–1.40)
	< 26	159	42 (26.4)	1 (reference)
High school education or higher	Yes	297	100 (33.7)	1.57 (1.23–1.99)
	No	428	92 (21.5)	1 (reference)
Social class	I-II	65	20 (30.8)	1.18 (0.80–1.74)
	III-IV	660	172 (26.1)	1 (reference)
Race	Non-white	18	3 (16.7)	0.62 (0.22–1.76)
	White	707	189 (26.7)	1 (reference)
Marital status: married	Yes	638	181 (28.4)	2.24 (1.27–3.95)
	No	87	11 (12.6)	1 (reference)
No. of previous pregnancies	> 1	135	28 (20.7)	0.67 (0.46–0.97)
	1	260	62 (23.8)	0.77 (0.59–1.01)
	0	330	102 (30.9)	1 (reference)
**Employment outside home**	Yes	351	91 (25.9)	0.96 (0.75–1.22)
	No	374	101 (27.0)	1 (reference)
**Cig/d smoked before pregnancy**	> 20	73	4 (5.5)	0.12 (0.05–0.31)
	11–20	347	47 (13.5)	0.29 (0.22–0.39)
	< 11	303	141 (46.5)	1 (reference)
**Partner's smoking**	Yes	508	110 (21.7)	0.57 (0.44–0.72)
	No	217	82 (37.8)	1 (reference)
**Change in alcohol consumption during pregnancy**	Quit/reduce	321	90 (28.0)	1.07 (0.83–1.38)
	Equal	80	17 (21.3)	0.81 (0.51–1.28)
	Do not drink	324	85 (26.2)	1 (reference)
**Year of the study period**	> 1999	380	76 (20.0)	0.52 (0.40–0.69)
	1999	174	51 (29.3)	0.77 (0.57–1.04)
	1998	171	65 (38.0)	1 (reference)
**Kessner index**	Adequate	262	93 (35.5)	1.82 (1.12–2.95)
	Intermediate	386	84 (21.8)	1.12 (0.68–1.83)
	Inadequate	77	15 (19.5)	1 (reference)

The independent variables related to smoking cessation yielded by a forward stepwise logistic regression model are summarized in table 4. All the variables detailed in table 3 were allowed to be in the model. Five significant predictors were included in the model. The number of cigarettes/day smoked before pregnancy was inversely related with cessation; the higher the number, the lower the frequency of cessation. The same occurred with three other variables: partner's smoking status, to have had a previous pregnancy, and the calendar year of the study period. Adequacy of prenatal care favoured smoking cessation. The addition of other variables to the model did not change any estimate of the former variables.

**Table 4:** Independent variables related to smoking cessation yielded by logistic regression analysis.

**Variable**	**OR (95% CI)**	***P*-value**
Cig/d smoked before pregnancy (continuous)	0.85 (0.82–0.87)	< 0.001
Kessner index (ref. inadequate):		
intermediate	1.14 (0.55–2.36)	0.718
adequate	2.72 (1.30–5.68)	0.008
Partner's smoking (ref. no)	0.57 (0.38–0.87)	0.010
No. of previous pregnancies (continuous)	0.75 (0.60–0.94)	0.014
Year of the study period (continuous)	0.77 (0.68–0.89)	< 0.001

## Discussion

We first comment on the limitations of the study. We have relied on the information given by women; their answers were not validated by cotinine measurements; therefore some degree of misclassification must be assumed. In a Spanish study, the proportion of pregnant non-smokers with negative urine cotinine (negative predictive value) was 82.9% [11]. This proportion is lower than that found in an American study [12]. where 94.9% of women who denied smoking yielded no urine cotinine. In general, it is believed that pregnant women accurately report tobacco smoking, although some under-declaration occurs [11,12]. There is no reason to believe that misclassification bias changed during the study period, so we believe that our results on the evolution of smoking in our region are reliable.

Our results agree with the decreasing frequency of smoking in women reported in Europe [13]; nevertheless, our data confirm a high prevalence of smoking among women of childbearing ages, almost 40% in 2002. The prevalence of smoking among pregnant women in Cantabria, above 30% throughout the quinquennial period 1998–2002, is one of the highest in the Western world; for instance, this figure was 21.8% in Sweden in 1992 [4], 21.2% in Finland in 1990 [14], 11.8% in the US in 1996 [5], and 4.4% in Czech Republic in 1997 [15].

The decreased frequency of smoking during pregnancy observed in Cantabria, 16.7% from 1998 to 2002, is higher than that found in other countries. In Sweden from 1983 to 1992 this figure was 7.6% [4], and 4.5% from 1987 to 1996 in the US [5] (although most of this decrease was observed for the period 1987–1990) [7]. The mean number of cigarettes/day smoked by pregnant women in Cantabria shows a slight trend to increase, although this amount is lower than the US figure, 10 cigarettes/day [5].

Our data show that the decline in smoking among pregnant women from 1998 to 2002 in Cantabria was primarily due to the overall decrease in smoking before pregnancy, not to an increased rate of smoking cessation in pregnancy; this has also been reported in the US [5]. Our figure of smoking cessation is lower than that found in other Spanish report, where 46.9% of smokers quit tobacco in pregnancy [16].

Regarding the determinants of smoking cessation, one of the most important factors is the intensity of habit at the beginning of pregnancy, being heavy smokers more reluctant to quit smoking [6,16-22]. Other predictor of smoking cessation frequently reported is partner's smoking status [17-19,21,23]. We also found this relationship in the multivariable model.

Primiparous women quit smoking more frequently than multiparous [23-27]. This is also supported in our data, where an inverse linear relationship between the number of previous pregnancies and smoking cessation has been observed.

In the US white race has been found a strong predictor of smoking in pregnancy [5,21,26-28]. In our sample the number of non-white women is small, thus we lack statistical power to draw any significant conclusion.

A high education level is negatively related with smoking [5,19,21-23,29], not found in one report [28]. In our data, a high education level increased smoking cessation in crude analysis, although this variable lost its statistical significance in multivariable analysis. Social class was unrelated to smoking cessation; in Denmark, this variable exerted only a small effect on cessation [19]. Married women showed a higher smoking cessation rate, but it lost its influence in multivariable analysis. This agrees with the results reported in other study [5].

We have only found one report relating adequacy of prenatal care to smoking cessation [6], women attending prenatal care in the first trimester quit smoking more successfully. This agrees with our results, women receiving adequate prenatal care (which by definition begins in the first trimester) show a higher rate of smoking cessation. This may also be due to personal characteristics of women: those prone to quit smoking attended more to prenatal care.

Some of the variables related to smoking cessation are not amenable by a prenatal care programme and need to be addressed by community programmes on quit smoking; this occurs with smoking before pregnancy or partner's smoking. We included in multivariable analysis the year of the study period, as a decline in smoking cessation along the study period was observed in bivariate analysis. This was done to ascertain whether other variables could take this influence into account. The effect of the year of study period remained highly significant in the multivariable model. It is important to remark that prenatal care improved considerably in Cantabria from 1998 to 2002. This was accompanied with a lower cessation rate. In Cantabria it is established in the written program of prenatal care to give advice on smoking cessation, but "how" to counsel is not detailed. In general, Spanish physicians are not trained in health education; this means that intervention on smoking cessation is low. To achieve changes in smoking behaviour among pregnant women with low-intensity interventions integrated into routine prenatal care is difficult [30]. It is assumed that counseling on smoking cessation did not change during the study period, what implies that Cantabrian pregnant women at the beinning of the 2000s are more reluctant to quit smoking than previously. This emphasizes the need to change the strategy of addressing smoking cessation in our region. Two systematic reviews have established the efficacy of smoking cessation programmes [31,32]; the following interventions are effective: a brief cessation counseling session of 5–15 minutes delivered by a trained provider, plus the provision of pregnancy specific, self help materials [32], or the Agency for Health Care Policy and Research guideline recommendations on smoking cessation [33].

## Conclusion

In Cantabria (Spain) a high frequency of smoking during pregnancy has been found, associated to both a decreasing smoking cessation and an improvement of prenatal care over time. These latter facts imply the need for a different approach (within prenatal care) of health education against tobacco smoking in Cantabria. Other variables, such as previous pregnancies or marital status, cannot been managed by prenatal care programmes. Several determinants of smoking cessation (smoking prior to pregnancy, partner's smoking status) need to be addressed by community programmes.

## List of abbreviations

RR: relative risk

OR: Odds ratio

CI: Confidence interval

## Competing interests

The authors declare that they have no competing interests.

## Authors' contributions

SP carried out the statistical analyses and drafted the manuscript. RP-I and RP-I collected the data. JL helped in the design and data collection. MM helped to draft the manuscript and statistical analysis. MD-R conceived the study, and participated in its design and coordination, and helped to draft the manuscript. All authors read and approved the final manuscript.

## Pre-publication history

The pre-publication history for this paper can be accessed here:


